# Self-Powered Sensors: New Opportunities and Challenges from Two-Dimensional Nanomaterials

**DOI:** 10.3390/molecules26165056

**Published:** 2021-08-20

**Authors:** Eunkwang Lee, Hocheon Yoo

**Affiliations:** 1 MLCC Development Team, Samsung Electro-Mechanics, Maeyeong-ro 150, Yeongtong-gu, Suwon 16229, Korea; unisteklee@gmail.com; 2 Department of Electronic Engineering, Gachon University, Seongnam 13120, Korea

**Keywords:** two-dimensional nanomaterials, optical sensors, self-power, nanostructure devices, sensor devices

## Abstract

Nanomaterials have gained considerable attention over the last decade, finding applications in emerging fields such as wearable sensors, biomedical care, and implantable electronics. However, these applications require miniaturization operating with extremely low power levels to conveniently sense various signals anytime, anywhere, and show the information in various ways. From this perspective, a crucial field is technologies that can harvest energy from the environment as sustainable, self-sufficient, self-powered sensors. Here we revisit recent advances in various self-powered sensors: optical, chemical, biological, medical, and gas. A timely overview is provided of unconventional nanomaterial sensors operated by self-sufficient energy, focusing on the energy source classification and comparisons of studies including self-powered photovoltaic, piezoelectric, triboelectric, and thermoelectric technology. Integration of these self-operating systems and new applications for neuromorphic sensors are also reviewed. Furthermore, this review discusses opportunities and challenges from self-powered nanomaterial sensors with respect to their energy harvesting principles and sensing applications.

## 1. Introduction

Sensors are key ingredients of next-generation electronics in the Internet of Things (IoT) because they contribute to collecting essential signals. The development of various sensors, including photosensors [1,2,3,4], gas sensors [5,6], temperature sensors [7,8,9], and biosensors [10,11,12,13] is extensive, which accelerates innovation in new technologies such as the aforementioned IoT. To realize the desired sensing functions, detection performance, such as high sensitivity, robust immunity to noise, and fast response times, should be comprehensively improved [14,15,16,17]. As another important performance metric, low power consumption is becoming more and more prominent [18,19,20,21,22]. Modern sensors require miniaturization operating with extremely low power levels to conveniently sense various signals anytime, anywhere, and to show the information in various ways. Thus, technologies combined with desirable materials that can harvest energy from the environment as sustainable, self-sufficient, self-powered sensors are to be considered a promising but necessary direction [23,24,25,26,27].

Meanwhile, the emergence of two-dimensional (2D) nanomaterials has ignited considerable interest in their potential, owing to their atomically thin nature [28] and superior electrical and optical characteristics. These 2D nanomaterials, including graphene [29,30], hexagonal boron nitride (hBN) [31,32], and metal dichalcogenides (MX_2_) [33,34], have a layered structure based on strong in-plane bonds and weak out-of-plane van der Waals (vdW) force. A lot of effort with 2D materials, including hBN insulators, molybdenum disulfide (MoS_2_) semiconductors [35,36], molybdenum diselenide (MoSe_2_) [35,36], tungsten disulfide (WS_2_) [37], tungsten diselenide (WSe_2_) [38], molybdenum ditelluride (MoTe_2_) [39,40], and black phosphorus (BP) [41,42], has been put into electrical, optical, and mechanical applications. High transparency resulting from the atomically thin nature and excellent charge transport ability from 2D crystallinity is expediting optoelectronic applications. Furthermore, the mechanical flexibility of these materials offers high compatibility, enabling wearable sensor systems. However, the extremely high voltage operation is considered a strong drawback, limiting sensor system miniaturization and wearable applications in conventional 2D nanomaterials–based sensors. In this light, the operation of self-powered sensors has rapidly emerged and has received a lot of attention in recent years [43,44,45,46,47]. Rather than use an external power source, self-powered sensors receive energy from the surrounding environment. Because there is a variety of environmental energy sources (e.g., piezoelectric [48], triboelectric [49], photovoltaic [50,51], and thermoelectric [50]), a combination of appropriate sensors and energy harvesting can implement a ubiquitous system without power consumption issues.

Here, an overview of self-powered sensors based on 2D nanomaterials is summarized. In Section 2.1, we provide an introduction to 2D nanomaterial-based photodetectors, and we revisit recent advances in their application to self-powered photodetectors. In Section 2.2, we discuss the fundamental principles of piezoelectric nanogenerators (PENG) for self-powered electronics, focusing on their potential applications. In Section 2.3, 2D nanomaterial-based triboelectric nanogenerators (TENG) are introduced. In Section 2.4, we discuss the thermoelectric effects of 2D nanomaterials with an emphasis on their application to self-powered thermoelectric devices. In Section 2.5, we revisit newly developed integration systems consisting of self-powered sensors. In Section 2.6, an emerging application of a neuromorphic self-powered system is introduced, presenting its merits from energy consumption aspects. In Section 3, we discuss the challenges and opportunities from future developments in 2D nanomaterial self-powered sensors.

## 2. Self-Powered Sensors

### 2.1. Self-Powered Photodetectors

Charge carriers (electrons and holes) in 2D materials are able to move quickly on a two-dimensional, quantum-confined surface within a thickness of several hundred nanometers. The emergence of graphene has boosted exploration into applications of 2D nanomaterials in photodetectors. Graphene is considered a promising optoelectronic material owing to its amenable energy-level tunability from a variety of chemical doping on its surface [51].

However, non–graphene-like 2D nanomaterials such as transition metal dichalcogenide (TMD), hexagonal hBN, and black phosphorus have gained attention from academia due to the diversity of their chemical compositions and structures. Investigating the phase-dependent properties is crucial to explore the 2D TMD applications. The phase and overall material properties of TMD are determined by the coordination of the transition metal and the stacking order between metal and chalcogenide atoms. Typically, the phase of TMD can be categorized with the terms 1T, 2H, and 3R. The number in each term denotes the different layers of the chalcogen atoms and transition metal in the c axis. On the other hand, T, H, and R, respectively, indicate tetragonal, hexagonal, and rhombohedral crystal symmetry. The properties of TMD are greatly affected by phase structure. For example, even for the same MoS_2_, the 2H phase represents the n-type semiconductor, and the 1T phase represents the metal. The electrocatalytic activity of 1T-MoS_2_ is much higher than that of 2H-MoS_2_ in hydrogen evolution reactions. Unlike 2H-MoS_2_, the bulk crystals of 1T-MoS_2_ exhibit superconductivity at 4 K. During the 2H-to-1T phase transformation of MoS_2_, the magnetism changes from diamagnetism to paramagnetism [52]. Typically, in chemical vapor deposition (CVD) processes, when a precursor reacts on substrate-rich dangling bonds, a film begins to grow from the dangling bonds. Since the original 2D TMD lacks dangling bonds as original reaction sites, the out-of-plane film growth is actually inhibited, and it grows in a plate-like shape. Atomically thick 2D TMDs have naturally passivated surfaces from the chalcogenide atoms. These 2D materials are chemically inert, making them suitable for incorporation into silicon structures without lattice mismatch issues [53].

Single-layer graphene generally does not have good light absorption, based on the zero band gap property. However, 2D TMD having a direct band gap is high in light absorption and can be implemented in high-performance optoelectronic devices, enabling the generation of excitons through photon illumination, suggesting a new kind of material [54]. TMD is a type of semiconductor with a wavelength of 590~1033 nm or an energy band gap range of 2800 K~4900 K and 1.2 eV~2.1 eV expressed in converted temperature and energy form. For example, MoS_2_ (1.8 eV), WS_2_ (2.1 eV), MoTe_2_ (1.1 eV), and WSe_2_ (1.7 eV) have a desirable band gap for optoelectronic devices [55]. These properties allow TMD-based photodetectors to identify both visible and NIR ranges, allowing them to be used in thrusters and active layer components in solar cells. In addition, to control the fine electrical properties of the TMD, its band gap can be adjusted in order to manufacture various electronic devices and long-distance temperature sensors in the NIR region by changing the number of layers of the TMD [56].

The utilization of 2D TMDs in photodetectors has been widely investigated. Overcoming technological hurdles, the development of self-powered photodetectors based on 2D TMD has been highly demanding, so far, for the following reasons. Self-powered photodetectors can work without external power sources. This advantage is beneficial for the fabrication of portable, wireless, and low-power devices. In addition, self-powered photodetectors can work independently, so a complex additional power system is not required. The combination of 2D TMD and a self-powered system would be of great advantage in the construction of lightweight, flexible energy-harvesting devices.

To evaluate the performance of a photodetector, we use various figures of merit, such as detectivity, photoresponsivity, dark current, etc. [57]. In particular, in order to achieve high detectivity (D*) in the photodetector, it is recommended to use a photosensitive material having an essentially low dark current. Almost all key parameters, such as D*, photoresponsivity (R), and photo-switching on/off ratio, are related to dark current. Traditionally, to suppress dark current, the photodetector (source, drain, and gate) based on the transistor structure is fabricated, and a gate bias is applied to control the charge carrier density of the channel. However, compared structurally to a two-terminal device, a transistor-type three-terminal device is more complicated to make and is inefficient because a gate bias must be continuously applied in terms of energy efficiency. Another way to achieve low dark current is to make a heterojunction TMD. Although this method can effectively improve the performance of the photodetector, it is still technically immature to make the heterojunction in the desired way (precisely and with a large area). Therefore, there is a need to develop a method that can encompass the aforementioned methods [58].

**Figure 1:**
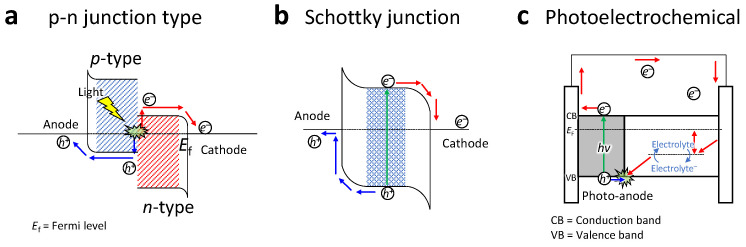
Self-powered photodetector/photovoltaic mechanisms: (**a**) the p-n junction type, (**b**) the Schottky junction type, and (**c**) the photoelectrochemical type.

Figure 1 exhibits several working mechanisms for the generation of photocurrent in photodetectors: the p-n junction type, the Schottky junction type, and the photoelectrochemical (PEC) type. The p-n junction and Schottky junction have similar mechanisms in which electron–hole pairs (e.g., excitons) are generated at the interface of the p-n type semiconductor for the p-n junction type (Figure 1a), or at the interface of a metal semiconductor for a Schottky junction, are separated by an applied electrical field (Figure 1b). However, in self-powered mode, spontaneously built-in electrical potential encourages separation of the excitons and drives them to the desired electrodes. On the other hand, the PEC type of mechanism encourages the excitons to separate toward anodes and cathodes via the energy barrier between the electrode and the electrolyte (as shown in Figure 1c). By using the PEC type structure, complete electron-circuit through the oxidation and reduction process in the electrolyte can be implemented in the self-powered PEC photodetector without consuming energy. Figure 1a shows an energy diagram where a space charge layer is formed due to the carrier concentration gradient at the junction when a p-type semiconductor and an n-type semiconductor are “junctioned” together and illuminated from the outside. An internal local electric field is formed in the p-n junction region, and when the light illuminates this part, the photogenerated electrons and holes move via the local electric field of the junction to generate a photocurrent [59,60].

In particular, various device structures of the p-n junction type in self-powered photodetectors have been reported. The basic structure of a p-n junction is a vertical junction in which two different 2D nanomaterials (p- and n-semiconductors) make contact. Meanwhile, a lateral junction can be fabricated during the artificial growth of 2D nanomaterials. Typically, heterogeneous reactants are introduced during 2D nanomaterial growth using chemical vapor deposition. Another approach to fabricating photodetectors is the fabrication of a mixed dimensional junction. For example, 1D nanowires or 3D bulk materials can be applied to make a unique junction. Those introduced approaches (vertical, lateral, 2D + 1D, and 2D + 3D junctions) are utilized for specific purposes (performance, flexibility, transparency, ease of fabrication, etc.)

**Figure 2:**
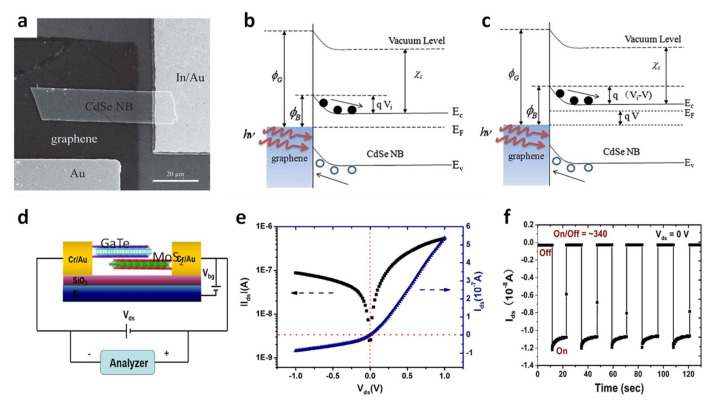
(**a**) FESEM image of a photodetector; (**b**,**c**) are energy band diagrams for the photodetector under external light irradiation of short circuit and load, respectively. Φ_G_ is the work function of graphene, Φ_B_ is the Schottky barrier height, V_i_ is the built-in potential, V is the voltage drop of the resistor, E_C_ is the conduction band edge, E_V_ is the valence band, and E_F_ is the Fermi level [61]. Reproduced with permission from [61]. Copyright 2012, Royal Society of Chemistry. (**d**) A schematic of a GaTe-MoS_2_ heterostructure phototransistor. (**e**) Current-voltage curve of the device (Vds = −1~1 V, V_bg_ = 0 V). (**f**) Self-driven photo-switch behavior of the GaTe-MoS_2_ heterostructure photodetector (V_ds_ = 0 V) [62]. Reproduced with permission from [62], Copyright 2016, American Chemical Society.

Jin et al. reported the fabrication of self-powered high-performance photodetectors using CdSe nanobelt (NB)/graphene, as shown in Figure 2a [61]. Graphene and Au are utilized as heterogeneous metal electrodes in the photodetector system to form a Schottky junction with CdSe NB. The difference in work function between CdSe (4.2 eV) and graphene (4.6 eV) will result in spontaneous built-in electrical potential. Energy band diagrams in a short circuit and with an external resistor are depicted in Figure 2b,c, respectively. When photon energy greater than the band gap energy of CdSe NBs is irradiated, the excited holes and electrons are separated by the built-in field and move to graphene and CdSe NB sides, respectively. The high photoresponsivity (10.2 AW^−1^) and gain (28) of the self-powered CdSe NB/graphene photodetector were obtained. The vertical junction of different 2D nanomaterials creates an unprecedented different charge transport behavior.

Yang et al. reported a self-driven photodetector using van der Waals nanomaterials MoS_2_ and GaTe, which are n- and p-type semiconductors, respectively. A GaTe-MoS_2_ phototransistor is shown in Figure 2d. A type-II band alignment is made in the GaTe-MoS_2_ junction by energy level alignment. In addition, huge built-in potential forces separation of excitons (electron and hole pairs) resulting in self-driven photocurrent. The I-V behavior of vertically stacked GaTe-MoS_2_ heterostructures (Figure 2e) shows typical forward bias rectification effects. The I-V behavior obtains an on-off current ratio of about 10. At the positive bias, the built-in potential at the interface between the two materials is greatly reduced, showing a high current. Conversely, at the negative bias, the built-in potential increases, and the off-current becomes smaller. Figure 2f shows a self-driven operation of the GaTe-MoS_2_ heterostructure at the zero bias (source-to-drain) under light illumination. The dynamic behavior of source–drain current is repetitive and maintained for a long period of time [62].

**Figure 3:**
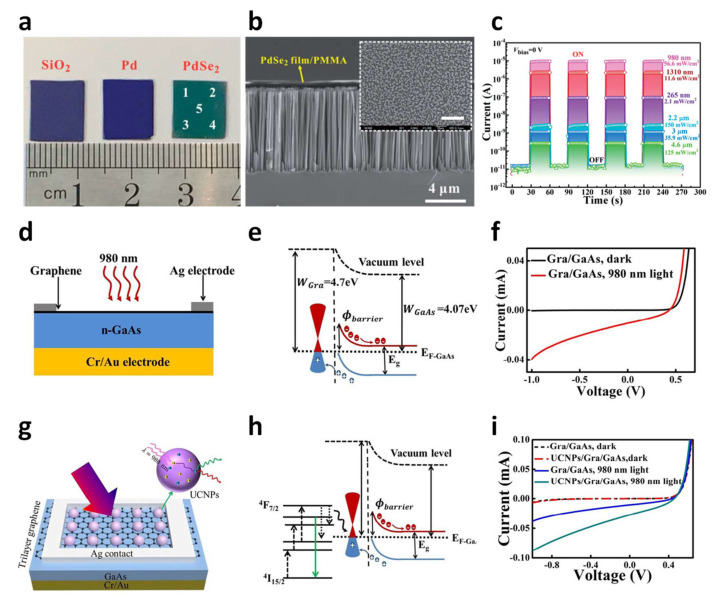
(**a**) Photographs of the SiO_2_/Si substrate, a Pd film, and a PdSe_2_ film formed on the SiO_2_/Si substrate. (**b**) Cross-sectional SEM image of a PdSe_2_ heterostructure vertically stacked on SiNWA. The inset shows a top view image of the heterogeneous structure. (**c**) Time vs. photocurrent plot of PdSe_2_/SiNWA heterostructure devices under illumination at different wavelengths (from 980 nm to 4.6 μm) at V_bias_ = 0 V. Reproduced with permission from [63]. Copyright 2020, Royal Society of Chemistry. (**d**) A cross-section view of a graphene/GaAs photodetector. (**e**) Electronic band alignment of the graphene/GaAs heterojunction. (**f**) I-V behavior of the graphene/GaAs photodetector in the dark (black) and under illumination from a 980 nm laser (red). (**g**) Top view of a graphene/GaAs photodetector coated with UCNPs. (**h**) Electronic band diagram of the UCNP/graphene/GaAs structure. (**i**) I-V behavior of graphene/GaAs or UCNP/graphene/GaAs photodetectors in the dark and under illumination from a 980 nm laser. The light illumination power is 55 mW. Reproduced with permission from [64]. Copyright 2018, Royal Society of Chemistry.

Not only the 2D–2D nanomaterial junction, but the construction of 1D–2D and 3D–2D vdW junctions has advantages from several aspects: (i) enhanced light absorption, (ii) a broadened spectral range from UV to IR, (iii) formation of built-in potential, which assists in separating photo-generated excitons, and (iv) more functionality. Wu et al. reported a self-driven, polarization-sensitive, broadband photovoltaic detector by transferring 2D PdSe_2_ onto the top of vertically aligned 1D Si nanowire arrays (SiNWA) [63]. Figure 3a is a photograph of an SiO_2_ substrate, Pd film on the SiO_2_, and PdSe_2_ film grown by direct selenization of the Pd film. The prepared PdSe_2_ film covered by polymethyl methacrylate (PMMA) was transferred to the SiNWA substrate to form a mixed-dimensional vdW heterostructure. Figure 3b is a cross-sectional SEM image of the PdSe_2_/SiNWA heterostructure. A thin film of PdSe_2_ is placed vertically on top of well-aligned SiNWA. Photocurrent can be detected via on/off light irradiation in various spectral ranges from deep ultraviolet (DUV) to midrange IR (Figure 3c). In particular, the fabricated PdSe_2_/SiNWA photodetector showed a self-powered characteristic that can be detected even when the voltage bias is 0 V. A stable, repeatable, and fast photoresponse from the self-powered photodetector was obtained, showing on/off current ratios (Ion/Ioff) of 10^4^ for 265 nm (2.1 mW cm^−2^), 10^6^ for 980 nm (56.6 mW cm^−2^), 10^2^ for 2.2 mm (150 mW cm^−2^), 3 mm (35.9 mW cm^−2^), and 26 for 4.6 mm (125 mW cm^−2^). Thus, the broad photo sensing range of the PdSe_2_/SiNWA heterostructure enables high-performance remote sensing, imaging sensors, and night vision.

Wu et al. fabricated a graphene/GaAs near-infrared photodetector (NIRPD) decorated with upconverting nanoparticles (UCNPs, NaYF_4_:Yb^3+^/E^r3+^) that can sense a light wavelength of 980 nm [64]. Figure 3d is a schematic of the graphene/GaAs NIRPD consisting of multilayered graphene, n-type GaAs, and Au and Ag electrodes. Figure 3e is an electronic band diagram of the graphene/GaAs. The work function (W_GaAs_) of heavily n-doped GaAs (1 × 10^18^ cm^−3^) is close to its electron affinity (χ = 4.07 eV). Graphene that shows a p-doped characteristic under an ambient condition has a work function of 4.7 eV. Therefore, when they come into contact, electrons diffuse from GaAs to the graphene to reach equilibrium in the electronic concentration gradient, which causes the GaAs energy band to bend upward at the heterojunction interface, with built-in potential created by this. As a result, at the interface of graphene and GaAs, the photo-excited carrier splits into an embedded electrical field under light illumination, generating an increasing reverse current, as shown in the I–V characteristic curve (the red line, Figure 3f). On the other hand, the graphene/GaAs NIRPD shows good rectification behavior in the dark (the black line). Figure 3g is a schematic of the UCNPs/graphene/GaAs NIRPD structure. UCNPs dispersed in an organic solvent are coated on the graphene surface. Figure 3h is an electronic band diagram of a graphene/GaAs NIRPD with UCNPs on the graphene surface. As shown in the energy band diagram, there are three mechanisms involved in an enhanced photosensing characteristic. The first is radiative energy transfer (RET). Due to the presence of UCNP, upconversion luminescence from the UCNP results in multiple photon pumping with low energy. The second mechanism related to the enhanced photosensing behavior is non-radiative RET. Absorbed light directly creates electron excitation in graphene. Lastly, an antireflection effect caused by UCNPs reduces the reflectance, which accompanies an efficient collection of light. *I*-*V* curves of graphene/GaAs and UCNPs/graphene/GaAs photodetectors in the dark and under illumination from a 980 nm laser are compared in Figure 3i. In dark conditions, both graphene/GaAs and UCNPs/graphene/GaAs photodetectors exhibit electrical current as low as 2.75 × 10^−10^ A in a grounded state. Contrarily, clearly increased reverse bias photocurrent for graphene/GaAs and UCNPs/graphene/GaAs photodetectors was obtained under illumination at 980 nm. Compared to a graphene/GaAs photodetector, UCNPs/graphene/GaAs photodetectors show greatly increased short circuit current by a factor of 2.6 times.

**Figure 4:**
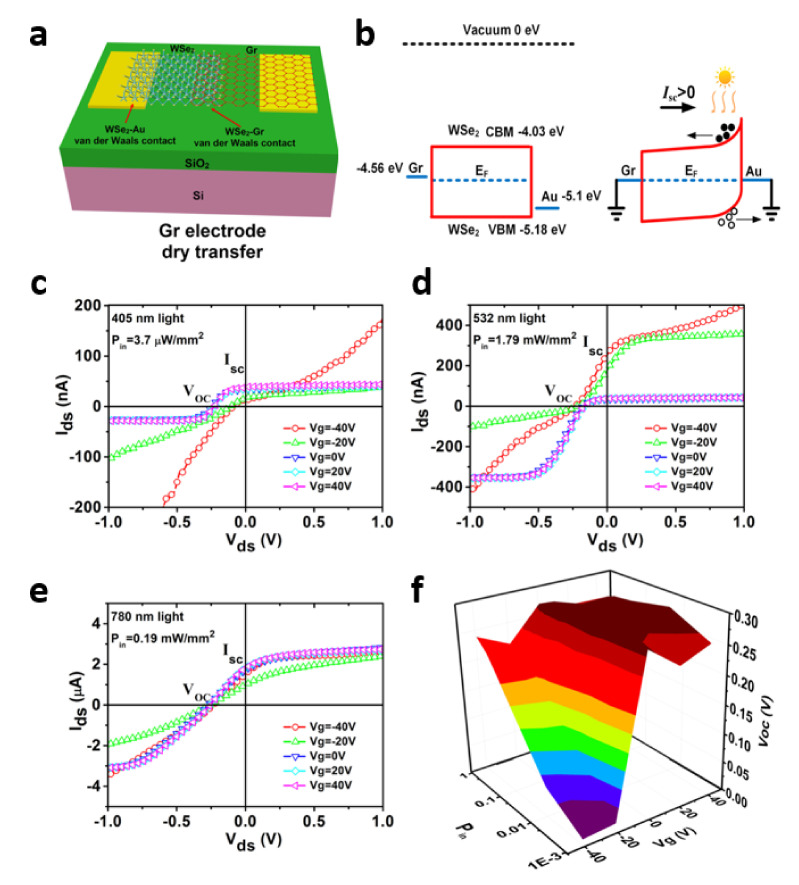
(**a**) A simple illustration of a graphene-WSe_2_-Au photodetector. (**b**) Energy band diagram of a device composed of graphene-WSe_2_-Au. Graphene and Au were used as electrodes. Current–voltage characteristics of graphene-WSe_2_-Au photodetectors are shown at wavelengths of (**c**) 405 nm, (**d**) 532 nm, and (**e**) 780 nm. (**f**) Dependence of the open-circuit voltage (Voc) on gate voltage bias and light power density at 780 nm illumination. Reproduced with permission from [65]. Copyright 2020, Springer Nature.

Zhou et al. reported an asymmetrical metal–semiconductor–metal (MSM) photodetector by using graphene and Au as the electrode contacting the WSe_2_ flake. The graphene and WSe_2_ were vertically stacked in part to form the van der Waals contact via the dry-transfer method [65]. Figure 4a is an illustration of a graphene-WSe_2_-Au photodetector on a SiO_2_/Si wafer substrate. As shown in the illustration, graphene and WSe_2_ are superimposed on each other in the middle. Figure 4b shows an energy band diagram of the photodetector with a graphene-WSe_2_-Au metal–semiconductor–metal (MSM) structure in the presence of light. It shows a large Schottky barrier difference between graphene and Au. In particular, in order to avoid surface material defects that can be induced through the process, and unavoidable doping and Fermi level pinning at the interface, a PDMS-assisted dry transfer method was utilized without the use of a metal deposition system. The WSe_2_ flake was at first transferred onto the pre-patterned Au electrodes, and the multilayer graphene flake was sequentially transferred onto the WSe_2_ flake. The fabricated asymmetric photodetector composed of graphene–WSe_2_–Au has self-powered characteristics, as evidenced by the I_sc_ − V_oc_ curve under light illumination, at a variety of wavelength ranges (405, 532, 780 nm) as shown in Figure 4c–e. Figure 4f summarizes the dependence of V_oc_ and the corresponding light power density on the gate voltage bias under a 780 nm wavelength. In conclusion, the graphene–WSe_2_–Au photodetector demonstrated similar gate-tunability through the change of light power intensity.

**Figure 5:**
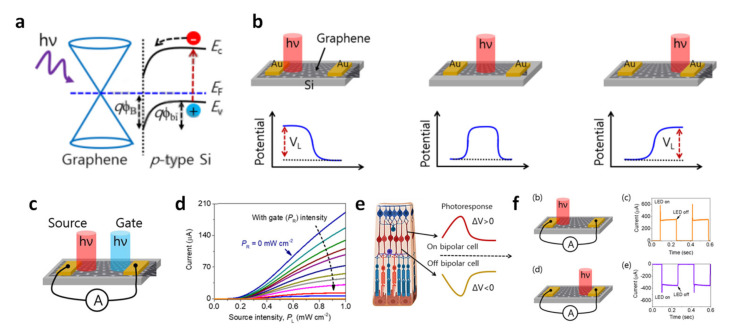
(**a**) Simplified energy band diagram of a graphene/p-type Si heterostructure. (**b**) The operational principle of the fabricated graphene/p-type Si device, and the location of light irradiation. (**c**) Illustration of a photon-triggered transistor (PTT) using two light sources. (**d**) The current–source light characteristic as a function of gate light intensity. (**e**) Functional schematic of the in vivo retinal information processing system. (**f**) A two-terminal device, illuminating the left- and right-electrode sides. The illumination conditions were λ = 420 nm, 2 mW cm^−^^2^. Reproduced with permission from [66], Copyright 2020, Elsevier.

Kumar et al. demonstrated a graphene/p-type Si-based electronic circuit modulated by a light illumination logic gate [66]. The graphene/p-type Si-based electronic circuit operates over a broad spectral range from ultraviolet to a near-infrared range under a zero bias. Figure 5a shows an electronic energy band diagram of the graphene/p-type Si-based electronic circuit. Due to the Fermi level pinning of graphene and p-type Si, and the formation of a Schottky barrier, energy band bending occurs at the interface between them, which might force the separation of excitons. According to the principle, a photon-induced electro-gating effect on the graphene/p-type Si can be observed on a selective area with a small spot size laser source, as shown in Figure 5b. Photon-induced potential can break the symmetry between the metal electrodes. Figure 5c is a schematic of a photon-triggered transistor (PTT). By modifying the appropriate driving source (light intensity, P_L_) and optoelectronic gate (P_R_) optical inputs, the PTT offers a way to inject the photogenerated carriers locally. Figure 5d shows the current–illuminance intensity (I-P_L_) curve as a function of light intensity from 0 to 1 mW cm^−2^. The device exhibits a current of up to 190 μA when powered only with light, and with no voltage applied. In particular, to mimic the human retina, we made an electronic device that mimics cells based on the voltage difference, as shown in Figure 5e, and that determines the polarity of the current according to the local location of the light. Thus, incident light near the left junction (P_L_ > 0 and P_R_ = 0) generates a current in both directions, as shown in Figure 5f. Conversely, when P_L_ = 0 and P_R_ > 0, the current changes in the negative direction.

### 2.2. Self-Powered Piezoelectric Devices

The acquisition of energy in our daily lives to run electronic gadgets is an intriguing issue when it comes to satisfying the energy needs of the Internet of Things (IoT). Nanogenerators are a promising platform for harvesting many forms of energy surrounding us. Nanogenerators can be related to vibrational energy, motion of the human body, mechanical triggering of tire rotation, wind flow energy, solar energy, blue energy, thermoelectric energy, and so on. Nanogenerators based on piezoelectric and triboelectric effects (PENG and TENG) are fascinating in this regard because they might combine several diverse nanogenerators into a single unit, which can utilize numerous energy sources simultaneously, enabling the use of any handy ambient energy at any time [67].

**Figure 6:**
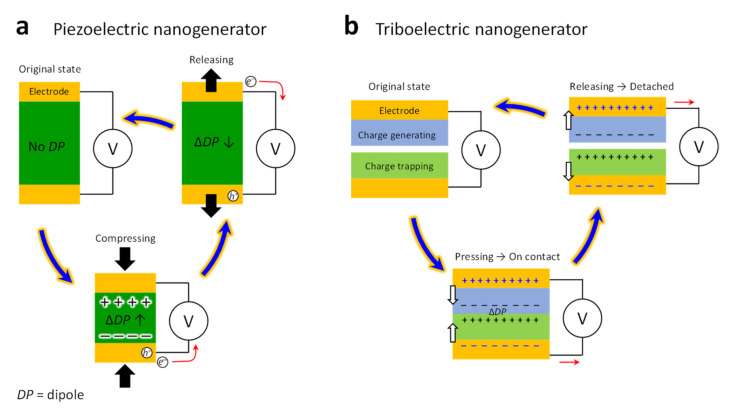
PENG- and TENG-based sensor mechanisms.

The mechanism underlying PENG operations is the direct piezoelectric effect. The piezoelectric effect is the electrical polarization of dielectric materials. In a direct piezoelectric mechanism, a force is applied along the asymmetric direction of the dielectric material, and a charge is generated on the opposite surface. When the applied force is removed, the dielectric material returns to its original state (Figure 6a) [68]. On the other hand, the fundamental operation of a TENG is a sequential state between contact and separation to realize the constant transfer of a charge, as shown in Figure 6b [67]. The charge-generating and -trapping materials make contact with each other, and dipoles are generated. Then, when the deformation is released and the materials detach, the opposite charges of the two surfaces will separate, so that these opposite triboelectric charges will generate an electric field between them, and thus, a difference of potential is induced between the top and bottom electrodes. Finally, the electrons will be driven to flow from one electrode to the other through the external load.

Interestingly, 2D monolayer nanomaterials have great potential in high-performance PENG and TENG. Unlike bulk materials, which are centro-symmetric structures, monolayer MoS_2_ is strongly piezoelectric under the strain-induced lattice distortion and charge polarization. Not only MoS_2_, but also a broad range of 2D materials, including TMDs, graphene, and monolayer group-IV and -III monochalcogenides, exhibit excellent piezoelectric effects depending on the PENG and TENG structure [69]. Regarding the fabrication of triboelectric devices, TENG performance can be expected to improve mainly from two aspects when 2D nanomaterials are used. When 2D nanomaterials are used, an improved induced charge generation rate can be expected, and the low internal resistance can contribute to an excellent output. In addition, the high specific area of 2D nanomaterials allows the formation of many charge-trapping sites [49].

**Figure 7:**
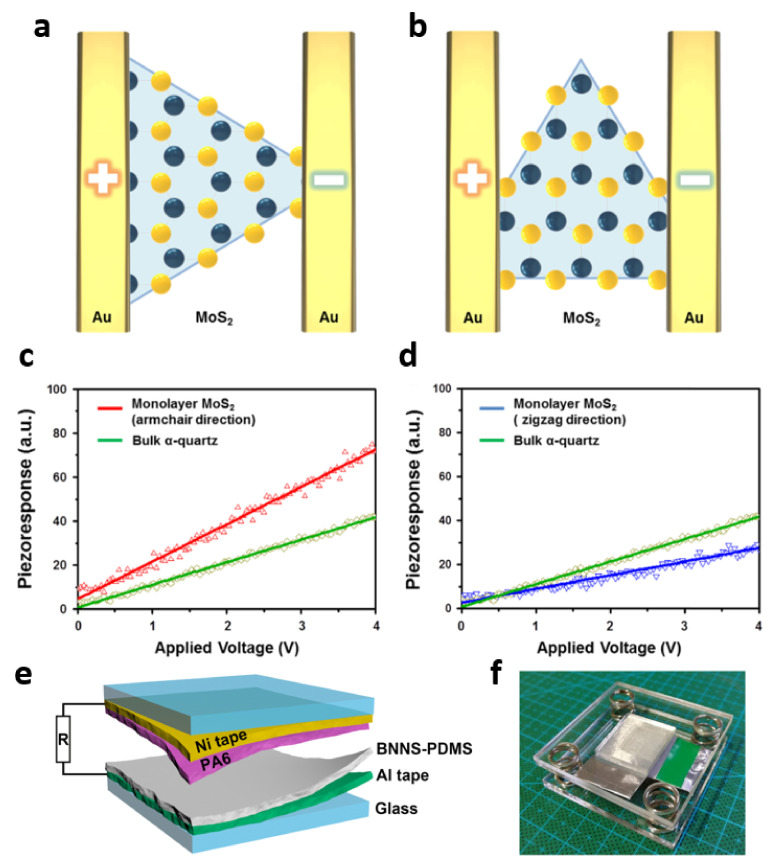
Corresponding piezoelectric modulus (d11) for monolayer MoS_2_ flakes and electrode positions in (**a**) the armchair direction and (**b**) the zigzag direction. High distinct piezoelectric response for monolayer MoS_2_ and α-quartz in (**c**) the armchair direction and (**d**) the zigzag direction. Reproduced with permission from [70], Copyright 2016, Elsevier. (**e**) A 3D schematic and (**f**) a photograph of the integrated PTEG device. Reproduced with permission from [71], Copyright 2020, Elsevier.

The piezoelectric power output from a single-layer MoS_2_ PENG is highly dependent on the atomic orientation. Kim et al. prepared MoS_2_ via the CVD method and confirmed that the piezoelectric modulus was different along the armchair (Mo and S parallel) and zigzag (Mo and S on the same line) directions (Figure 7a,b) [70]. Figure 7c shows a high distinct piezoelectric response (piezoelectric coefficient, 3.78 pm/V) for monolayer MoS_2_ and α-quartz, as a function of the voltage magnitude in the armchair direction, compared to α-quartz. In contrast, the piezoelectric response in the zigzag direction showed a lower response, compared to α-quartz, with a piezoelectric coefficient of 1.38 pm/V (Figure 7d).

The presence of piezoelectricity in multilayer hBN has been predicted theoretically, and the piezoelectric voltage coefficient of 2.35 × 10^−3^ VmN^−1^ has been experimentally confirmed. Applying this, Kuang et al. demonstrated the piezoelectric effect of the hBN nanosheet (BNN)-PDMS composite membranes [71]. The schematic device structure and photos of the BNNS-PDMS/PA6 PTEG are shown in Figure 7e,f. Thus, a piezoelectric and triboelectric nanogenerator (PTEG) was fabricated by grafting a PA6 membrane onto a BNNS-PDMS membrane.

**Figure 8:**
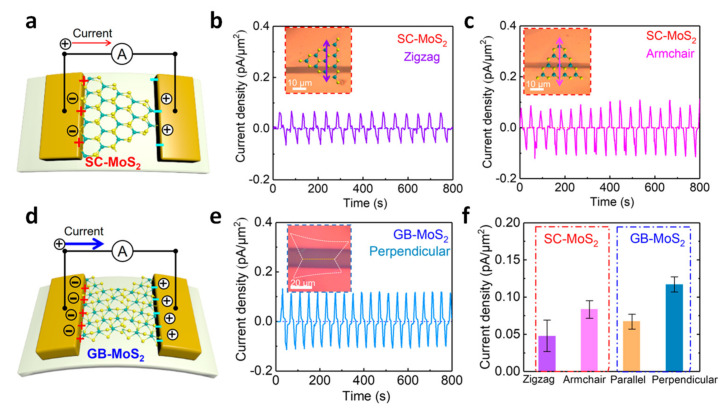
(**a**) Illustration of the SC-MoS_2_ flake-based flexible piezoelectric device. The real-time current output of SC-MoS_2_ piezoelectric devices along (**b**) zigzag and (**c**) armchair orientations. (**d**) Illustration of a GB-MoS_2_ flake–based flexible piezoelectric device. (**e**) Current output obtained from the GB-MoS_2_ piezoelectric device perpendicular to the grain boundary direction. (**f**) Current densities of MoS_2_-based piezoelectric devices under various conditions under the same experimental conditions. Reproduced with permission from [72], Copyright 2020, American Chemical Society.

Dai et al. investigated the morphological structure of grain boundaries (GBs) in MoS_2_ flakes and observed the improved piezoelectric effect through the existence of the GBs [72]. The measured piezoelectric output power of the MoS_2_ with GB (GB-MoS_2_) exhibited 50% higher than a single-crystal MoS_2_ (SC-MoS_2_). The piezoelectric response was recorded as shown in Figure 8a–d. The output current density of SC-MoS_2_ with a zigzag orientation is displayed in Figure 8b, showing that the current density induced by deformation of the device is about 0.05 pA/μm^2^. On the other hand, the output current density of SC-MoS_2_ with an armchair orientation is displayed in Figure 8c, showing that the current density induced by deformation of the device is about 0.08 pA/μm^2^, which indicates that the armchair orientation of MoS_2_ results in a slightly higher value for current density. As for the GB-MoS_2_–based device shown in Figure 8e, the current densities are about 0.07−0.12 pA/μm^2^—much larger than from the SC-MoS_2_. The statistical results of current density were obtained from those devices, as shown in Figure 8f. In conclusion, when more GB is present in MoS_2_, it shows a higher current output result, indicating that the contribution of piezoelectric enhancement by GB is significant.

### 2.3. Self-Powered Triboelectric Devices

**Figure 9:**
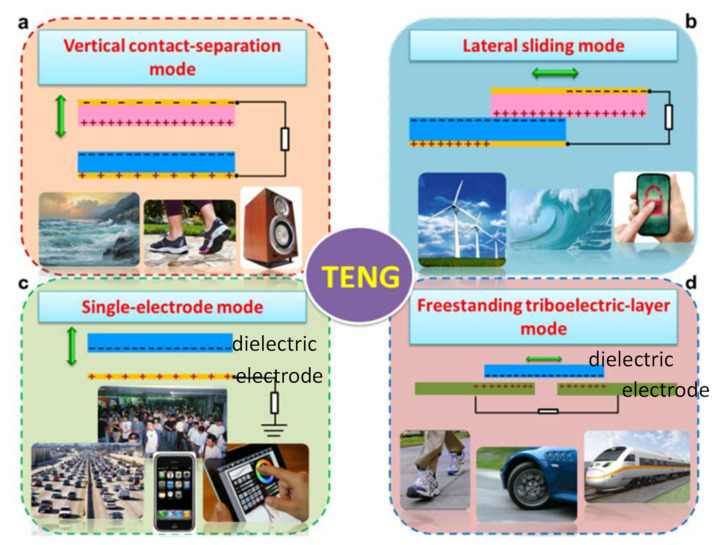
Four basic modes of operation in triboelectric nanogenerators: (**a**) vertical contact separation mode, (**b**) side-sliding mode, (**c**) single-electrode mode, and (**d**) standalone mode. Reproduced with permission from [73], Copyright 2015, Royal Society of Chemistry.

The triboelectric effect is a type of electrification induced by contact between different electrostatically charged materials. Recently, TENGs have received lots of attention from academia and industry due to an increased interest in renewable energy that can convert available mechanical energy into electrical energy. Figure 9a is the simplest form of TENG. It works when contact between two different dielectrics occurs, and electrodes are deposited at both ends to extract electrons generated by friction. Physical contact between these structures creates opposing charged surfaces in a vertical manner. Figure 9b has the same structure as Figure 9a, but when two dielectric films come into contact, they generate frictional charges through a sliding motion in the lateral direction. Lateral sliding begins to flow in an external circuit to balance the field generated by the triboelectric electrons. In this case, AC output is generated via periodic rubbing from side to side. For fabrication of a simpler structure, single-electrode TENG can be fabricated, which consists of a bottom electrode and an active component (Figure 9c). In the single-electrode system, if the distance between the electrodes changes, the electrical potential is disturbed. Then, electrons will be transported in order to balance electrical charges. On the other hand, certain dielectric layers can be established on top of two separated metal electrodes without contacting each other. Moving the dielectric material back and forth, asymmetric charge distributions are generated, which might cause an electrical current flow (Figure 9d). The oscillation of the electrons caused by moving the dielectric produces electrical power. In this case, the freestanding dielectric layer does not need direct contact. A rotational TENG device can be designed without any mechanical contact between the dielectric and metal electrodes Thus, it is a good approach to enhancing the durability of the nanogenerator.

**Figure 10:**
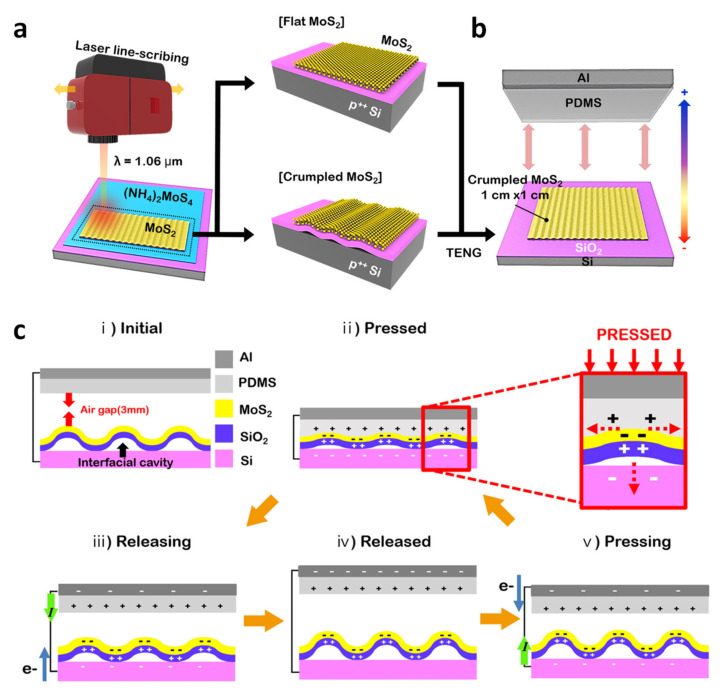
(**a**) Illustration of laser-induced synthesis of 2D MoS_2_ on the SiO_2_/Si wafer. (**b**) Illustration of a PDMS-MoS_2_–based TENG device structure. (**c**) Five steps of the mechanism in the fabricated TENG device. Reproduced with permission from [74], Copyright 2020, Elsevier.

Park et al. characterized TENG devices consisting of either flat or crumpled MoS_2_ layers. The crumpled MoS_2_ was synthesized by laser-directed thermolysis [74]. Figure 10a is a schematic for fabrication of the flat and crumpled MoS_2_ using a laser line-scribing machine on an (NH_4_)_2_MoS_4_–coated Si wafer substrate. The photothermal interaction induced by the laser creates sufficient heating of the (NH_4_)_2_MoS_4_ film, which forms a 2D MoS_2_ layer. Interestingly, if the irradiation intensity of the laser is stronger than 2.62 Jcm^−2^, the crumpled structure of the MoS_2_ layer is formed. As illustrated in Figure 10b, either the flat structure or the crumpled structure of the 2D MoS_2_ is utilized to fabricate a contact-separation type TENG to figure out the nanostructure effect of 2D MoS_2_ layers. PDMS, which is a commercial silicone elastomer, is used as a counterpart triboelectric material. As shown in Figure 10c, in crumpled MoS_2_, two operating principles are involved: contact charging and electrostatic induction. In the initial state (i), there is an air gap between the MoS_2_ and the Al/PDMS composite electrode, so that no electron flow occurs, and in (ii), when contact between the two layers occurs, a triboelectric charge is formed on the surface due to electron transfer from PDMS to the MoS_2_. Compared to the less crumpled and most crumpled MoS_2_, the most crumpled MoS_2_ results in a greater shear friction effect on the device. On the other hand, a flat MoS_2_ produces almost no shear friction. When a negative triboelectric charge is generated on the most crumpled MoS_2_ surface, the PDMS surface is converted to a positively charged state. Thereafter, as we separate the Al/PDMS electrode from MoS_2_, in (iii), the potential within the separation generates a current from the MoS_2_ side to the Al/PDMS electrode side throughout the external circuit to compensate for the friction charge generated by electrostatic induction. When fully released and returned to the original state, in (iv), the balanced charge does not generate any current. When a mechanical force is again applied to the device, as seen in (v), a friction charge is again formed on the surface and the principle seen in step (ii) is repeated.

**Figure 11:**
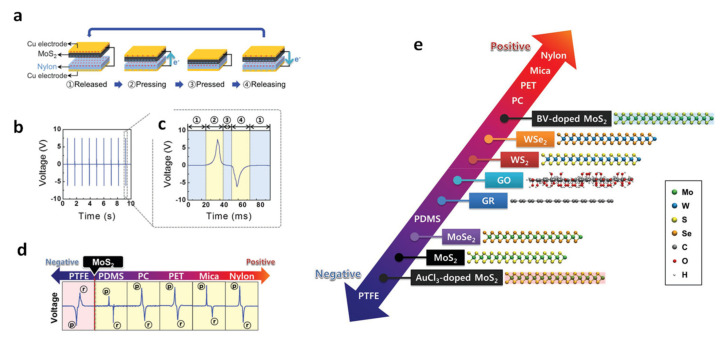
Triboelectric behavior of MoS_2_ and several polymer films: (**a**) the device structure and working principle of MoS_2_-nylon TENG, (**b**) the output voltage signal, and (**c**) its output voltage signal in one cycle. (**d**) Triboelectric behavior of various polymer film-MoS_2_ combinations. (**e**) The modified triboelectric series with 2D materials. Reproduced with permission from [75]. Copyright 2018, John Wiley and Sons.

Seol et al. investigated the triboelectric charging behaviors of various 2D materials to understand separate working mechanisms in TENG. They used commercially available 2D nanomaterials, such as MoS_2_, MoSe_2_, WS_2_, WSe_2_, GR, and graphene oxide (GO), which were chemically exfoliated from their bulk materials. Then, a thin film of the 2D nanomaterials was prepared using a vacuum filtration method. Figure 11a is a schematic showing the MoS_2_–nylon TENG device structure consisting of two pairs of Cu electrodes. Figure 11b shows the output voltage characteristics for the MoS_2_–nylon TENG. There are typically four distinctive parts in the output characteristics, as shown in Figure 11c. The designated numbers (①–④) in the plot of Figure 11c matches the released, pressing, pressed, and releasing steps depicted in Figure 11a. In particular, a charge transfer is induced by physical contact between MoS_2_ and the nylon (see Figure 11(a③)). After contact, releasing the two materials induces a reversed electrical current in the external circuit between the two Cu electrodes. Thus, a negative voltage is observed at ④ in Figure 11c. It determines that MoS_2_ is negatively electrified when contacted by nylon. Then, a positively induced voltage is observed in the MoS_2_–nylon TENG device, which indicates that the transferred electrons transport in the reverse direction through the external load (see Figure 11(a②)). In a similar manner, various TENGs were fabricated using MoS_2_ and commercially available polymer films, as shown in Figure 11d. From Figure 11d, it is possible to quantify the degree of triboelectric behavior, depending on which base material is used. The output value is measured during repeated press and release functions. Interestingly, TENGs with MoS_2_-PTFE films show negative output during the press, but positive output during the release. This shows that MoS_2_ becomes anodized after contact with PTFE. Conversely, PDMS, PC, PET, and mica exhibit cathodic properties. By documenting this, we can summarize the triboelectric series as shown in Figure 11e for several two-dimensional materials and polymer films. MoS_2_ had the most negative triboelectric charging performances than other 2D materials (MoS_2_, MoSe_2_, graphene, graphene oxide, WS_2_, WSe_2_) and all the 2D materials were placed at the negative side of the triboelectric series, compared to the studied polymeric films (PC, PET, mica, and nylon). Importantly, the synthetic method and the thickness of MoS_2_ are barely associated with charging polarities. Instead, the charging characteristics of the triboelectric effect in MoS_2_ could be strongly affected by chemical doping to modify the work.

### 2.4. Self-Powered Thermoelectric Devices

**Figure 12:**
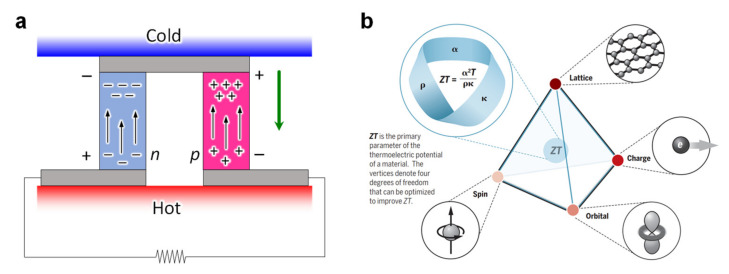
(**a**) A schematic of a thermoelectric sensor. (**b**) Thermoelectric materials research is a multidisciplinary study involving charges, spins, orbitals, and lattice degrees of freedom in matter. Studies into electrical resistivity (*ρ*), the Seebeck coefficient (*α*), and thermal conductivity (*κ*) should be conducted simultaneously. Reproduced with permission from [50], Copyright 2017, American Association of the Advancement of Science.

Heat and electricity are very intimate forms of energy in daily life. However, the applicability of heat and electricity is quite different. Heat can be found everywhere around us, but it is difficult to change it to a more useful form in a thermodynamic way, whereas electricity is more practical for powering electrical appliances, but electricity is not ubiquitous and must be stored. Thermoelectrics provides a direct solution for converting heat to electricity in an eco-friendly way [50]. For example, a thermocouple can generate electrical current to operate processes directly, without the need for extra circuitry and power sources. Thus, the power from a thermocouple can trigger a valve when a temperature difference arises. In a thermocouple, continuous heat from the hot side generates the electrical energy to maintain the electric potential between hot and cold sides, in which the Peltier effect dominates to drive the electrical charge in the circuitry. A thermal flow occurs to lower the temperature of the hot side, thereby causing electrical flow. Therefore, it is important to drive the device to allow continuous heat flow. A thermoelectric device produces a voltage when there is a temperature difference between the hot and cold sides. On the other hand, when a voltage is applied to the thermocouple, it produces a temperature difference. Figure 12a is a schematic of a typical thermoelectric sensor. At the macroscopic scale, an applied temperature gradient force charges the carrier in the material, diffusing it from the hot to the cold side, which generates electricity in the sensor circuitry. In addition, an exact temperature, or a change in the temperature of objects, can be measured by reading electrical current amperage. The term thermoelectric effect implies three effects separately: the Seebeck effect, the Peltier effect, and the Thomson effect. The Seebeck effect is a potential difference generated in a closed circuit due to a temperature difference. The Peltier effect is a temperature difference that occurs when one side is exothermic and the other side is endothermic when a potential difference is applied. The Thomson effect refers to an exothermic or endothermic phenomenon that occurs when an electric current flows and there is a partial temperature difference in the same metal. For example, the negative Thomson effect is when current flows from a low-temperature region to a high-temperature region, and it becomes endothermic (for example, Pt, Ni, or Fe). The positive Thomson effect is when a current flows from a high temperature region to a low-temperature region, and it becomes exothermic (for example, Cu and Sb). Thermoelectrics is currently looking for specialized niche applications in the 21st century. In particular, although the current research trend places a lower priority on efficiency than energy availability and reliability, research on availability and reliability is required. Specifically, it is necessary to develop high-performance materials for the wide application of thermoelectric devices in the future. Because thermoelectric materials generate electricity with a change in temperature, the dimensionless figure of merit, ZT = *α*^2^T*ρ*^−1^*κ*^−1^, can be calculated from the Seebeck coefficient (*α*), electrical resistivity (*ρ*), and thermal conductivity (*κ*). In this regard, for manufacturing high-performance thermoelectric devices, high ZT is an important criterion for determining material performance. Therefore, in order to improve ZT, it is necessary to optimize electrical resistance, the Seebeck coefficient, and thermal conductivity. Achieving high material performance in terms of the microscopic atomic-scale level comes with delicate balances between the phase stability and instability, structural order and disorder, bond covalence and ionicity, band convergence and splitting, transversal and local electronic states, and trade-offs between carrier mobility and effective mass (Figure 12b).

**Figure 13:**
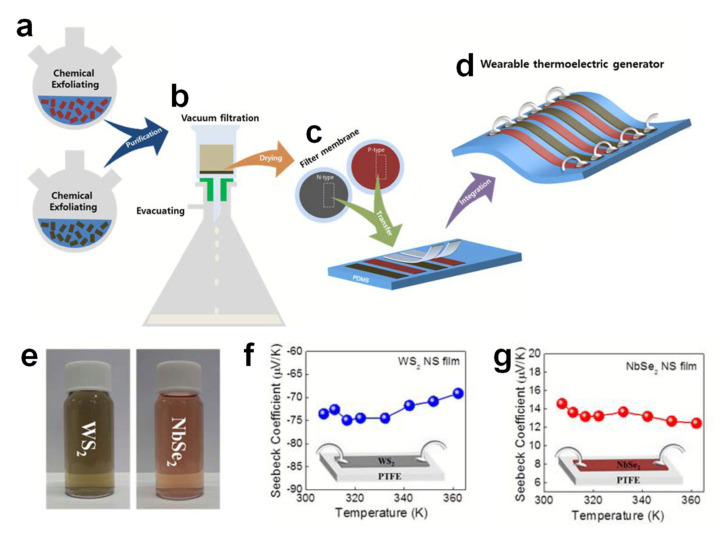
Chemically exfoliating TMD nanosheets and manufacturing a wearable thermoelectric generator using TMD nanosheets: (**a**) preparation of chemically exfoliated TMD, (**b**) vacuum filtration of TMD nanosheets using a membrane filter, (**c**) transfer of a vacuum-filtered TMD nanosheet film to a PDMS substrate using a contact printing method, and (**d**) the connection between Ag wire and thermoelectric film using Ag paste. (**e**) WS_2_ and NbSe_2_ dispersed in aqueous solution, With graphical representations of thermoelectric properties from 300 K to 360 K for chemically exfoliated (**f**) WS_2_ and (**g**) NbSe_2_. Reproduced with permission from [76], Copyright 2016, Royal Society of Chemistry.

Oh et al. fabricated a two-dimensional nanosheet-based flexible thermoelectric device that can obtain energy at room temperature from body temperature [76]. Through chemical exfoliation in a solution process method, a large amount of TMD nanosheets that can be used in the manufacture of thermoelectric devices were synthesized. Figure 13a–d are illustrations showing the process such as the preparation of chemically exfoliated TMD nanosheet films to fabricate wearable thermoelectric generators. A solution can be prepared that separates the bulk TMD material into single/several layers of 2D nanosheets through a chemical exfoliation process. There are two steps in this process. The first step is to insert Li ions into the interlayers of the bulk TMD. n-Butyl lithium (Bu-Li) molecules migrate into octahedral sites per interlayer of the bulk TMD, and Li atoms and TMD meet to form a LixTMD compound from their high chemical affinity. Next, Li ions of the LixTMD compound react with DI water to form LiOH, and the lamellar TMD is peeled off. This reaction causes the interlayer space of the TMD to expand, weakening the van der Waals force, and can easily be exfoliated by sonication. This chemically peeled TMD solution can be vacuum filtered through a membrane to produce the desired TMD thin film (Figure 13b). The TMD film produced in this way can be patterned into the desired shape, and TMD-PDMS thermoelectric devices can be fabricated through the contact printing method (Figure 13c,d). To fabricate a thermoelectric generator, n-type and p-type TMD films must be prepared and fabricated on a PDMS substrate. Figure 13e shows WS_2_ (n-type) and NbSe_2_ nanosheet (p-type) solutions. The WS_2_ film used as the n-type thermoelectric film exhibits the highest Seebeck coefficient of approximately −70 to −75 μVk^−1^ (Figure 13f) and high conductivity (approximately 1.0 to 1.4 × 10^3^ Sm^−1^) at room temperature. On the other hand, p-type NbSe_2_ shows about +12~+14 μVk^−1^ for the Seebeck coefficient, and about 150~170 × 10^3^ Sm^−1^ in conductivity, as shown in Figure 13g. A wearable thermoelectric generator consisting of those 2D TMD nanosheets can be used to generate electric energy in a self-powered way from the human wrist through the thermoelectric generator.

### 2.5. Self-Powered Sensor Integration

**Figure 14:**
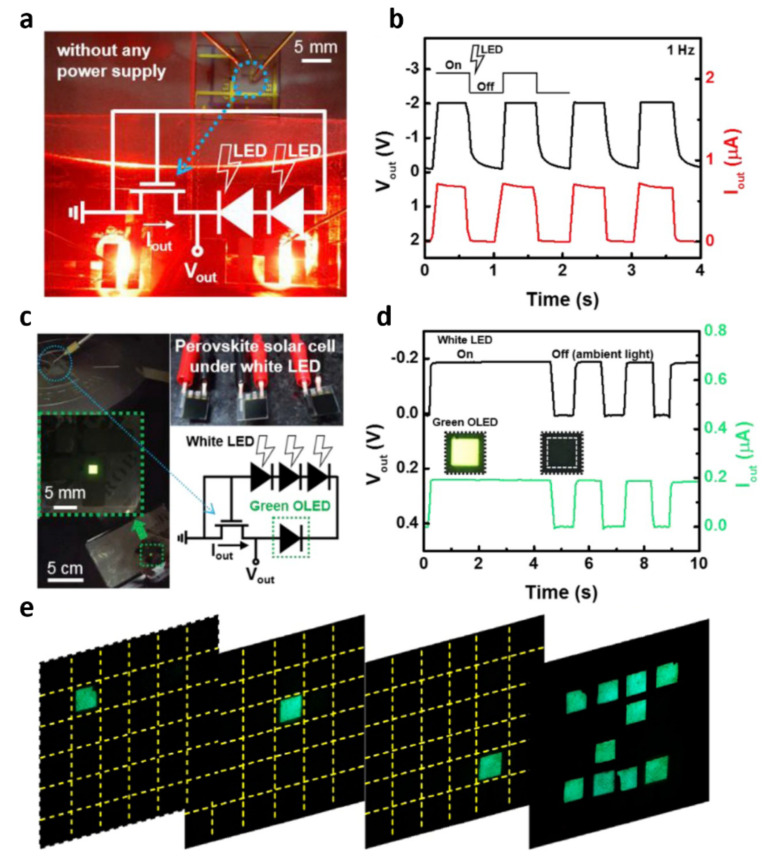
Self-powered integrations: (**a**) optical image of the MoTe_2_ transistor and perovskite photovoltaic cells connected in series under red LED illumination, and (**b**) pulsed measurement of an integrated circuit under a red LED pulse at 1 Hz; (**c**) integration of green OLEDs combined with perovskite photovoltaic cells and MoTe_2_ switching transistors, and (**d**) pulsed measurement of green OLED under white LED switching. Reproduced with permission from [77], Copyright 2019, Elsevier. (**e**) Pattern array image of MXene-based alternating current electroluminescence integration. Reproduced with permission from [78], Copyright 2021, Elsevier.

The aforementioned 2D material–based self-powered sensors offer extremely low power consumption, and integrated applications have been developed. A self-powered circuit integration of perovskite cells and 2D MoTe_2_ transistors was successfully demonstrated by Im et al. in 2019 [77]. Methylammonium lead iodide perovskite photovoltaic cells connected with MoTe_2_ transistors provided a dynamic response under a red light pulse at 1 Hz (Figure 14a,b). Furthermore, they implemented photovoltaic-powered green organic light-emitting diodes (OLEDs), presenting pulsed green light illumination according to white light–source switching behaviors (Figure 14c,d). From remarkable progress in 2D materials–based self-powered sensor integration, a TENG integrated array using MXene was demonstrated by Shan et al. in 2021 [78]. An alternating current electroluminescence (ACEL) array connecting TENG device integrations led to a self-powered pattern display. The flexible and transparent 4 mm^2^ ACEL display arrays successfully exhibited pattern Z (Figure 14e).

**Figure 15:**
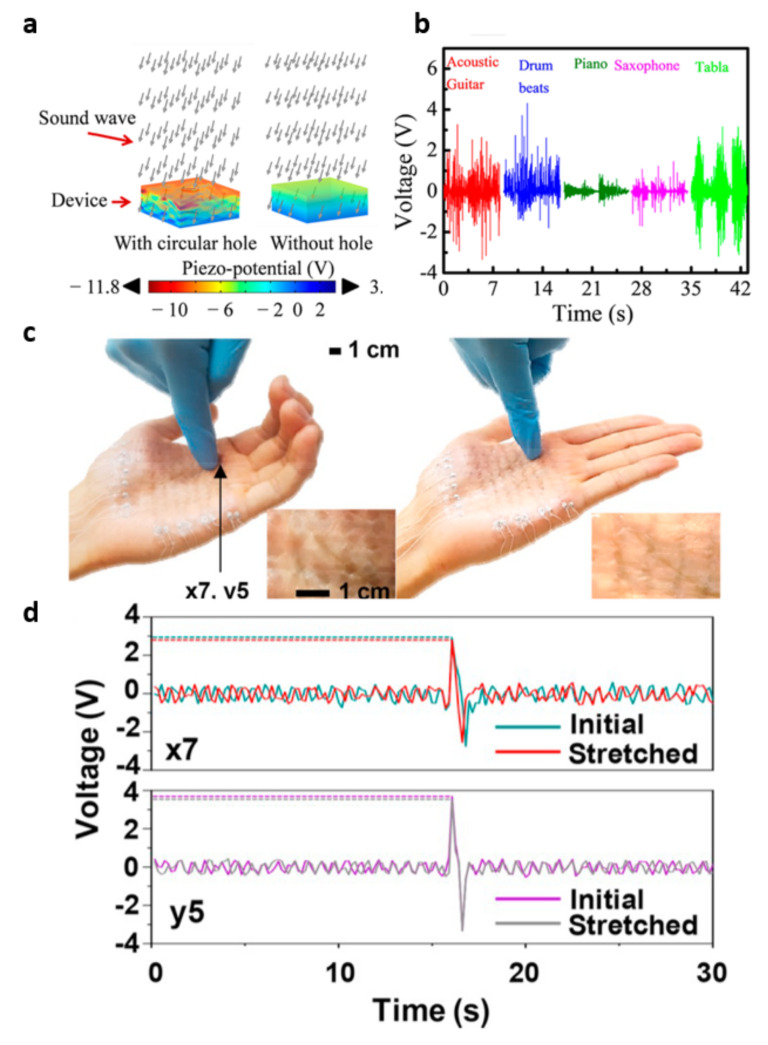
Self-powered sensor integrations: (**a**) a piezo-potential distribution analysis of acoustoelectric conversion and (**b**) output voltage characteristics conducted with different musical instruments under that sound pressure level. Reproduced with permission from [79], Copyright 2021, American Chemical Society. (**c**) TENG touch sensor integration on the palm of the hand, and (**d**) output voltage measurement before and after stretching. Reproduced with permission from [80], Copyright 2019, Elsevier.

In 2021, a 2D metal-organic framework (MOF) and polyvinylidene fluoride (PVDF)-based piezoelectric nanogenerators were presented by Sinha and Mandal et al. [79]. In addition to the excellent piezoelectric output at as high a power density as 24 µW·cm^−2^, an acoustoelectric application was demonstrated (Figure 15a). At a 110 dB acoustic vibration, successful acoustoelectric conversion was performed, exhibiting energy-harvesting behavior from 120 Hz at 110 dB for five blue light-emitting diodes. Furthermore, the proposed acoustoelectric conversion was demonstrated with five musical instruments (guitar, drum, piano, saxophone, and the tabla) (Figure 15b). As another interesting application, a self-powered stretchable TENG-based touch sensor was presented by Ahn et al. [80]. From atomically thin graphene integrated with a polyethylene terephthalate substrate and a poly-dimethyl siloxane electrification layer, a highly stretchable TENG array was fabricated. Touch-sliding velocity was successfully detected through the graphene-based stretchable TENG sensors, providing self-powered operation without an energy source.

### 2.6. Self-Powered Neuromorphic Applications

**Figure 16:**
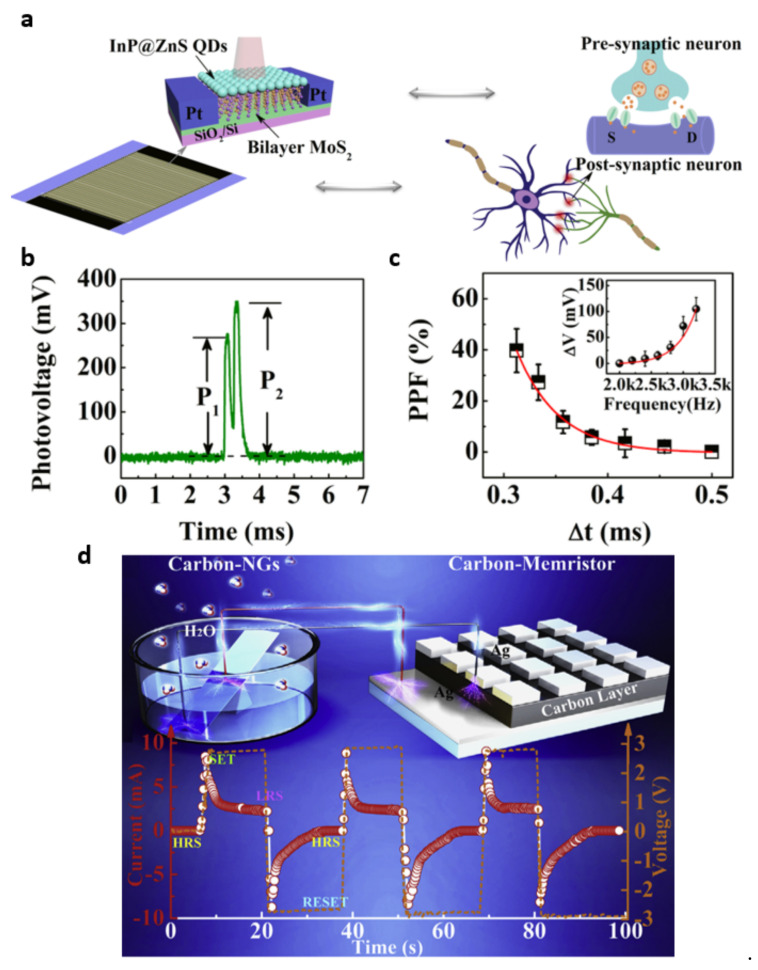
Self-powered neuromorphic systems: (**a**) the InP@ZnS-MoS_2_ hybrid phototransistor and a neural network with a multiple series−parallel synapses schematic, (**b**) paired-pulse facilitation (PPF) induced by two laser pulses under a 532 nm laser at 170 mW·cm^−2^, and (**c**) dependence of the PPF on the interval between paired pulses (Δt) from a 532 nm laser at 170 mW·cm^−2^; the inset shows the variance of the photovoltage as a function of pulse frequency from 2000 to 3200 Hz. Reproduced with permission from [81], Copyright 2020, American Chemical Society. (**d**) A self-powered system using carbon-NGs cells connected in series with a memristor. The air velocity is controlled at ~1.0 ms^−1^. The output current versus the time for the carbon memristor after integrating it with three carbon-NGs cells shows the expected voltage output. Reproduced with permission from [82]. Copyright 2019, Elsevier.

The emergence of artificial neural network hardware facilitates the development of electronic devices mimicking the brain. These neuromorphic devices potentially obtain extremely low–power computing, and fast decision-making, processes. In this context, the concept of self-powered neuromorphic applications targets self-driven artificial neuroprocessing with respect to the architecture as well as the physical energy source. In 2020, Rosei et al. presented mixed dimensional (0D/2D) phototransistors with excellent photoresponsivity as high as 1374 A·W^−1^ under self-powered operation [81]. Using the proposed phototransistors, they also demonstrated artificial synaptic behaviors, such as excitatory postsynaptic and paired-pulse facilitation, opening up the development of the self-driven neuromorphic system (Figure 16a–c). In addition, a self-powered nanogenerator–based memristor was proposed [82]. Connecting amorphous carbon nanogenerators to the memristor demonstrated self-powered nanogenerator–memristor cells, exhibiting set/reset-based low resistance–state (LRS) and high resistance–state (HRS) switching behaviors (Figure 16d).

## 3. Conclusions

Various examples of self-powered 2D-nanomaterial sensors offer many benefits, as revisited in this paper. However, further advances are required for practical application development. The 2D materials-based sensors still have challenges, and there are many directions for future research. The following requirements should be addressed: (1) develop a large-area synthesis method for 2D nanomaterials, (2) establish a reliable phase control engineering process, (3) secure long-term reliability and stability, and (4) implement process development that is compatible with flexible substrates.

First, to realize an integrated, self-powered system at more than a sensor array level, large-area synthesis technology with a high yield must be developed. The current technology status is still implementation at the flake level or the centimeter level containing defects or grain boundaries. To make 2D nanomaterials available for real sensor systems, a uniform and robust materials platform should be developed. Second, the 2D-material phase control process must be further developed. Optoelectrical properties of 2D nanomaterials significantly depend on their crystalline phase, such as 1T, 2H, and 3R. The material needs to be adjusted to make it compatible with the function of each electronic sensor element. Third, in order to gather big data and to be used in the IoT, long-term stability should be secured. Still, previously reported sensor devices have shown a lack of long-term stability evaluation. It is necessary to introduce a new evaluation method to realize a sensor for long-term continuous healthcare monitoring based on self-powered technology. Fourth, mechanical flexibility in plastic substrate process development should be accomplished. For wireless technology and healthcare sensors, wearable devices are essential. To maximize the potential benefits of 2D nanomaterials, compatibility with flexible substrates is needed. As mentioned, self-powered 2D-nanomaterial sensors still have difficulties in the synthesis and fabrication processes, and with reliability. However, the advantages and possibilities from their functionality are expected to help us envision autonomous wearable sensors.
